# Detection of vaccine-derived poliovirus type 2 from sewage samples and public health response, Poland, November to December 2024

**DOI:** 10.2807/1560-7917.ES.2025.30.1.2400805

**Published:** 2025-01-09

**Authors:** Magdalena Wieczorek, Beata Gad, Arleta Krzysztoszek, Paulina Kłosiewicz, Kinga Oleksiak, Bartosz Zaborski, Paweł Grzesiowski, Katarzyna Tkaczuk, Anna Baumann-Popczyk

**Affiliations:** 1 Department of Virology, National Institute of Public Health NIH – National Institute of Research, Warsaw, Poland; 2 Bureau of Research and Technology, MPWIK SA (Municipal Water Supply and Sewerage Company in Warsaw), Warsaw, Poland; 3 The Chief Sanitary Inspector, Chief Sanitary Inspectorate, Warsaw, Poland; 4 Department for Communicable Disease Prevention and Control, Chief Sanitary Inspectorate, Warsaw, Poland

**Keywords:** cVDPV2, polio virus, environmental surveillance, NIE-ZAS-1 emergence group

## Abstract

In October and December 2024, circulating vaccine-derived poliovirus type 2 (cVDPV2) was detected from two wastewater samples in Poland during routine environmental surveillance. The first isolate was characterised and matched previous cVDPV2 isolates detected in Spain in September, as well as in Germany, Finland, and the United Kingdom in November and December 2024. In response to the event, active surveillance for acute flaccid paralysis (AFP) has been strengthened, and the frequency of environmental sample collection has been increased.

Since September 2024, circulating vaccine-derived poliovirus type 2 (cVDPV2) has been detected from wastewater samples within routine environmental surveillance (ES) in five European countries [1], including Poland, where the virus was isolated from a sewage sample in Warsaw in October and in Rzeszów in December. Here we describe the events, the investigation and the public health measures taken.

## Environmental surveillance of poliovirus in Poland

In 2024, wastewater samples were routinely collected from eight sampling sites in six cities in Poland (Warsaw, Lublin, Rzeszów, Kraków, Gdańsk, Wrocław), at the inlets to wastewater treatment plants. In Warsaw, three samples were taken: two from the largest wastewater treatment plant in Poland Czajka and one from Południe. In the other cities, one sample was taken per sampling session (Figure). Samples were collected twice a month.

**Figure:**
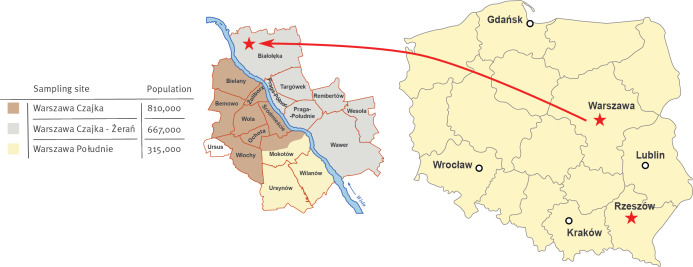
Map presenting sites for environmental surveillance of poliovirus, Poland, 2024

The red stars indicate the geographic location of the detection of poliovirus type 2 from a wastewater sample in Warsaw and Rzeszów.

The wastewater samples were analysed using a method described by Zurbriggen [2], a method applied in Poland since 2011, followed by isolation in cell cultures, as recommended by the World Health Organization (WHO) [3]. Two cell lines are used: L20B (5 flasks) and RD (1 flask). Positive cultures in the L20B cell lines undergo intratypic differentiation (ITD) analysis to identify the virus type and the origin of the virus (wild or vaccine). All poliovirus isolates are then sent to the Regional Reference Laboratory of the WHO/Europe for Poliomyelitis, Robert Koch Institute (RKI) in Berlin, for confirmation and sequencing of the VP1 coding region of the capsid protein.

## Detection of poliovirus type 2 in Warsaw

From 1 January to 3 December 2024, 74 wastewater samples were collected in Warsaw. Of these, 73 tested positive for non-polio enteroviruses (NPEVs) (NPEV detection rate: 98.6%). Polioviruses were identified in 4 of 74 samples (5.4%). Vaccine virus, Sabin-like poliovirus type 3 (SL3), was detected from samples collected in January, July and September (Table). Circulating vaccine-derived poliovirus type 2 (cVDPV2) was detected from a wastewater sample collected on 22 October, from one of six inoculated flasks. The L20B-positive culture was confirmed using real-time reverse-transcription PCR in the ITD assay version 6.0. The new oral polio vaccine type 2 (nOPV2) assay, part of the ITD 6.0 kit and designed to detect the presence of the nOPV2, was negative.

**Table:** Sampling of wastewater in surveillance of poliovirus, Warsaw, Poland, 9 January–3 December 2024

Calendar week	Date	Sampling site					
		Czajka (CZ)	Czajka-Żerań (CZZ)				Południe (PD)
2	9 Jan	NPEV	NPEV				NPEV
4	23 Jan	NPEV and SL3	NPEV				NPEV
6	6 Feb	NPEV	NPEV				NPEV
8	20 Feb	NPEV	NPEV				NPEV
10	5 Mar	NPEV	NPEV				NPEV
12	19 Mar	NPEV	NPEV				NPEV
15	9 Apr	NPEV	NPEV				NPEV
17	23 Apr	NPEV	NPEV				Neg
19	7 May	NPEV	NPEV				NPEV
21	21 May	NPEV	NPEV				NPEV
23	4 Jun	NPEV	NPEV				NPEV
28	9 Jul	NPEV	NPEV				NPEV and SL3
30	23 Jul	NPEV	NPEV				NPEV
32	6 Aug	NPEV	NPEV				NPEV
34	20 Aug	NPEV	NPEV				NPEV
37	10 Sep	NPEV	NPEV				NPEV
39	24 Sep	NPEV	NPEV and SL3				NPEV
41	8 Oct	NPEV	NPEV				NPEV
43	22 Oct	NPEV	NPEV and cVDPV2				NPEV
45	5 Nov	NPEV	NPEV				NPEV
46^a^	14 Nov	NS	NPEV	NPEV	NPEV	NPEV	NS
47	19 Nov	NPEV	NPEV				NPEV
48^a^	26 Nov	NS	NPEV	NPEV	NPEV	NPEV	NS
49	3 Dec	NPEV	NPEV				NPEV

cVDPV2: circulating vaccine-derived poliovirus type 2; Neg: not detected; NPEV: non-polio enterovirus; NS: no sample; SL3: Sabin-like virus type 3.

^a^ Additional samples were collected directly from the sewer network in the four smaller catchment areas of Warsaw Czajka-Żerań.

The obtained isolate was sent to the RKI which confirmed the results and sequenced the VP1 coding region. The sequence differed by 44 nt from the Sabin 2 vaccine strain. Genetic analyses conducted by the WHO and the United States Centers for Disease Control and Prevention (US CDC) showed our isolate matched the viruses detected in routine surveillance of wastewater systems in Spain, Germany, Finland and the United Kingdom (UK) since September 2024 [4]. The genetic analysis linked the isolate to the circulating VDPV2 NIE-ZAS-1 emergence, which was first detected in Zamfara, Nigeria in 2020 and has since spread primarily in northwestern Africa [5]. This genetic connection of our Polish environmental isolate with strains circulating in Africa has placed Poland in the WHO category of an affected country [6].

## Detection of poliovirus type 2 in Rzeszów

On 11 December 2024, cVDPV2 was detected from a wastewater sample from Rzeszów, collected on 3 December. From 1 January to 3 December 2024, 23 sewage samples had been collected in Rzeszów, and all tested positive for NPEVs. An SL 3 isolate was obtained from a sample collected in July. The cVDPV2 virus was isolated from one L20B-positive flask of six inoculated flasks (5 L20B and 1 RD) and identified using the ITD 6.0 kit. This result was confirmed by the RKI. The sequence differed by 46 nt from Sabin 2 strain and matched the NIE-ZAS-1 cVDPV2 group recently detected in Europe.

## Public health response

The detection of cVDPV2 triggered the coordinated activation of the National Polio Preparedness and Response Plan. A rapid risk analysis was conducted, and active surveillance for acute flaccid paralysis (AFP) in Warsaw and Rzeszów region has been strengthened. Inspections are being carried out in paediatric hospitals, in neurological wards, to detect unreported AFP cases. A media campaign was launched to encourage the completion of overdue vaccinations of children. The sampling frequency in Warsaw has been increased to once a week, and new sampling points in the wastewater network have been designated in order to divide the monitored area into four smaller zones. New sampling dates were added to the previously established schedule which resulted in the Warsaw Czajka-Żerań catchment area being alternately monitored as one entire area (one sample) and four smaller zones (four samples). No other polioviruses have been detected in these subsequent samples (Table). Additional samples from Rzeszów were scheduled to be collected on 30 December 2024.

From 1 January to 3 December, 15 cases of AFP in children aged < 15 years were identified in the Masovian Voivodeship surrounding the capital of Warsaw and four cases in Subcarpathian Voivodeship. No human cases of polio have been reported. Based on the results obtained, the detection of cVDPV2 from an environmental sample in Poland was classified as a sporadic event, given the lack of evidence of virus transmission in the community.

## Discussion

Poland has been polio-free since 2002, as the entire WHO European Region. The last recorded case of polio in Poland was diagnosed in 1984 [7], before the launch of the Global Polio Eradication Initiative (GPEI) in 1988. Poland has used the oral polio vaccine (OPV) for the longest period in the European Union (EU), discontinuing its use in 2016 following the global switch from trivalent OPV (tOPV) to bivalent OPV (bOPV). Since then, in Poland, inactivated polio vaccine (IPV) is used, with three doses of basic vaccination administered at 3–4, 5–6 and 16–18 months of age and a booster dose given at the age of 6 years. Currently, polio immunisation coverage in Poland is > 86%, in Warsaw 82% and in Rzeszów 83% (level of polio vaccination for three doses of IPV vaccine). According to the report from the European Regional Commission for the Certification of Poliomyelitis Eradication (RCC), Poland is classified as at an intermediate risk of transmission following the importation of wild poliovirus or the circulation of VDPV, due to decreasing subnational immunisation coverage [8]. In the opinion of the Working Group for Epidemiological Surveillance and Risk Assessment of the Spread of Polio Viruses in Poland (data not shown), the detection of cVDPV2 in Poland – a country with high standards of hygiene, relatively high vaccination coverage and well-functioning environmental and clinical surveillance systems – suggests a low risk of virus spread within the country.

Poland conducts AFP surveillance. In 2011, ES was started when a large wastewater study was conducted, covering 13 cities [9]. From 2017 to 2020, a study was conducted on wastewater samples taken at Warsaw international airport to assess the risk of poliovirus importation into Poland via passenger air traffic [10]. In 2021, ES was established in Warsaw, and in subsequent years, it was expanded to other cities: Lublin and Rzeszów in 2022, Kraków and Gdańsk in 2023, and Wrocław in 2024. The expansion of ES nationwide was prompted by Poland receiving large numbers of war refugees from Ukraine.

The proportion of NPEV-positive sewage samples is an indicator of the functioning of ES. According to the WHO, at least 30% of concentrated sewage from grab samples should test positive for NPEVs [3]. The detection of NPEVs from samples collected in Warsaw and Rzeszów in 2024 was high (98.6–100%), indicating high effectiveness in virus recovery. Considering this high NPEV detection rate and the detection of cVDPV2, which has emerged in Europe, we consider that our ES setup is sensitive enough.

Despite the success in eradicating wild poliovirus types 2 and 3, detection of cVDPV of all types, particularly type 2, is a considerable global health issue. Between January 2023 and June 2024, 70 cVDPV2 outbreaks from 34 emergence groups were reported across 38 countries [11]. The NIE-ZAS-1 cVDPV2 emergence group has spread to countries mostly in northern and western Africa [12]. The detection of this group in Europe in 2024, initially in Barcelona, then in two locations in Poland and in other European countries, indicates intercontinental spread signalling gaps in current control measures and responses to emerging outbreaks in Africa. According to authors from WHO and the US CDC, larger and faster responses in Africa, including vaccination campaigns using type 2-containing OPV, will be necessary to stop further cVDPV2 transmission [13].

## Conclusions

The detection of cVDPV2 from wastewater samples in Poland underscores the need of continuous ES, also in areas considered polio-free. These findings highlight the importance of strengthening clinical surveillance and educating the medical community which may overlook the obligation to report AFP cases in regions widely considered polio-free. Furthermore, enhancing vaccinations campaigns should be developed and reasons for vaccine hesitancy be understood and addressed. Consideration may also be given to legal regulations that enforce vaccination compliance.
